# Physiology

**Published:** 1860-11

**Authors:** S. Weir Mitchell

**Affiliations:** Lecturer on Physiology in the Phila. Med. Association


### PHYSIOLOGY.

BY S. WEIR MITCHELL, M.D.,
LECTURER ON PHYSIOLOGY IN THE PHILA. MED. ASSOCIATION.

I.—*Note on the Coloration of the Bones of the Fœtus, by the Action of Madder mixed in the Food of the Mother.* By M. FLOURENS.

It is near twenty years since I presented to the Academy (3d February, 1840,) two or three skeletons of pigeons, reddened by the action of madder which had been mixed, for a certain time, with the food of those animals. The last experiments of this kind were made in France, by Duhamel, a century before mine, viz., in 1739. These had been almost forgotten; mine were received with curiosity by physiologists.

Passing from my observations on birds to those on mammals, I presented to the Academy, February, 24, 1840, the skeletons of two or three puny pigs whose bones and teeth were completely reddened by the action of madder mixed with the food.

To-day I present to the Academy a fact much more curious, and as I believe quite new. Not only are the bones of the animal nourished with madder, but those of the fœtus reddened and made of a much deeper color, merely by submitting the mother to a diet mixed with madder during the last forty-five days of gestation.

In this instance the bones and teeth have both alike become red, and are even more deeply tinted than happens in cases where young animals have been directly fed on madder. No other tissues than the bones share in the coloration to which the other parts of the fœtus are subjected, and the periosteum, the cartilage, and all the viscera remain of their normal colors.

M. Flourens exhibited specimens in illustration of the above statements, and made certain inferences as to the communication between the blood of the mother and that of the fœtus.—(*Comptes Rendus.*)

II.—At the recent meeting of the British Association for the Advancement of Science many physiological papers were read. The following brief notice of some of them is given by the *Lancet, July 9th*, 1860.

A paper was read by Dr. Edward Smith, in which the action of tea and that of alcohol were contrasted, embodying the results of various researches he had made on the subject. He proved that the consumption of tea is increasing in all parts of the world, that it is beneficial and nutritive, and that the consumption of alcohol is decreasing. He gave the effects of each on the system, and showed the difference between the two; also the conditions in which tea was applicable and inapplicable. He showed the essential differences between the effects of coffee and of tea on the system, and all the highly interesting experiments referred to were chiefly performed upon himself and another person. An animated discussion took place, in the course of which Mr. W. Milner, surgeon to the Wakefield Prison, detailed a series of experiments, made with permission, on the prisoners, concerning the use of tea and other substances. His observations went to prove the correctness of Dr. Smith's statement, that tea does diminish the weight of the body, unless given with solid food. Dr. Smith asserts that the use of tea increases the waste of the tissues.

Mr. Garner read a paper on Certain Alterations in the Medulla Oblongata in Cases of Paralysis. The author showed that atrophy of the columns of the medulla oblongata, in cases of continued paralysis, is not rare, and touched upon the probability of an arrangement in the spinal cord by which the separate action of the extensor and flexor muscles in tetanus, etc. is caused. He illustrated the intimate connection of the olivary bodies with the posterior part of the medulla oblongata by tearing it down longitudinally, when they will always be drawn out backward with that part. The author connected the olivaries with speech and respiration. The paper also described the varied insertion of the optic and olfactory nerves with various parts of the encephalon, according as they were associated with the intellectual or locomotive functions. He did not believe in any distinct separation of the upper and lower columns in the invertebrata.

Contributions to the Theory of Cardiac Inhibition formed the subject of a communication from Dr. Michael Foster. A series of experiments were detailed, illustrating the action of various degrees of intensity of the galvanic current upon the contraction of the heart, through the agency of the pneumogastric nerves. These experiments were chiefly confined to the snail, but the crab and other animals were likewise operated upon, with results proving the inhibitory action of the heart.

An Experimental Inquiry into the Mode of Death produced by Aconite, by Dr. E. R. Harvey, was next read. The author's experiments, which were performed on dogs, rabbits, and frogs, went to prove that aconite acts first on the nerves and then on the muscles, killing by its action on the heart; the blood and urine were found normal; Fleming's Tincture was the preparation used. Among the various speakers was Sir Benjamin Brodie, who referred to his own experiments, made many years back, in which he had used the expressed juice of aconite; it acted as a narcotic, and arrested respiration first, and he had no doubt that if artificial respiration had been tried, the animal would have recovered, as in cases of poisoning by woorara. It seemed to him not improbable that different preparations of aconite produce various effects. Dr. Sharpey observed that, in some former experiments he had made with aconite, he obtained the same results as Dr. Harvey, namely, the action of the heart was stopped and the irritability of the muscles was extinguished; the nerves also leading to the affected muscles were deprived of their excitability.

Dr. Gibb read an interesting paper on Saccharine Fermentation within the Female Breast, and its Influence on the Child. He showed that from various causes of a constitutional nature, in which the nervous system played an important part, the saccharine element of the milk underwent fermentation at the moment of its secretion, and gave rise to the generation of two species of animalcules, namely, vibriones and monads. The milk containing these was usually rich in sugar, but owing to the fact of its having undergone fermentation within the gland itself, its healthy character was destroyed and it was not therefore capable of assimilation within the stomach of the infant, as evidenced by the most extreme degree of emaciation—in fact, the child was undergoing starvation. The animalcules were developed within the breasts. The author had proved the correctness of his views, in a series of experiments and researches into this question since 1854. In the discussion which ensued much credit was given to the author for his labors in this novel field of inquiry, and numerous questions were put to him in relation to the condition of the blood and other fluids, in such conditions as he had described.

Professor Corbett read a paper on the Deglutition of Alimentary Fluids, in which he attempted to prove that the epiglottis did not always fall back upon the glottis during the swallowing of fluids. This was denied by some of the speakers, who thought it always did occur.

One of the most valuable papers of the physiological section, was an elaborate one from Mr. Arthur E. Durham, being An Experimental Inquiry into the Nature of Sleep. He described the appearance of the brains of animals, as seen through openings made in their skulls, and, supporting by a process of ingenious reasoning his hypothesis upon this most important subject, Mr. Durham stated that during sleep the brain was pale and comparatively bloodless; that during functional activity the pia mater and brain substance were highly injected and full of rapidly-moving blood. He suggested that the increased amount of oxygenated blood coursing through the vessels during wakefulness was the cause of the functional activity of the brain, and that the opposite state of the circulation gave rise to the cerebral inactivity of sleep. The state of sleep was accurately distinguishable from that occurring in all varieties of coma. Mr. Durham's views were ably supported by Professor Draper, of New York, but dissented from by Dr. Beale.

Dr. Beale demonstrated, by a series of preparations and diagrams, how every elementary fibre of striped muscle was abundantly supplied with nerves. He believes that every single fibre receives one or more nervous fibres, which will be found hereafter to account for complicated muscular movements. Wherever these minute nerves are distributed are found little oval bodies, which are the means of communication between the muscular and nervous fibres. These nerves are found to be more numerous in some muscles than in others—the tongue and diaphragm for example—and are surrounded by multitudes of these oval bodies. The nervous fibres are constantly undergoing decay and being reproduced. Dr. Beale's discovery is one of considerable value in physiology, and much importance was attached to it by the Association.

Dr. B. W. Richardson read a lengthy paper on the Influence of Oxygen on Animal Bodies. He enumerated a series of laborious experiments on the inhalation of oxygen. By these he established two major propositions:—1. That the influence of pure oxygen on animals varies according to the animal, being most marked in those of quick respiration and high temperature, and less marked in those of feeble respiration and lower temperature. 2. That oxygen gas, breathed over and over again, and freed entirely from any substances which we yet know as products of respiration, loses the power of supporting life, the vital process ceasing, not from the introduction of a poison, but as by a negation or a withdrawal of the vivifying principle.

The Physiological Relations of the Coloring Matter of the Bile was the subject of the next paper, by Dr. Thudichum. He had subjected various specimens of chlorochrome (as he proposed to term the substance) to chemical analysis, and found that it was an amido-acid, and nearly related to the other acids of the bile. Its precipitation from solution in bile, when it occurred already in the gall-ducts, by a process closely resembling that of putrefaction of bile in a bottle, was an ascertained cause of so-called idiopathic jaundice. He described various products of decomposition of chlorochrome; among them chlorochromic acid, a substance which microscopically closely resembled hæmatoidine.

